# Antibiotic-Related Adverse Drug Reactions at a Tertiary Care Hospital in South Korea

**DOI:** 10.1155/2017/4304973

**Published:** 2017-12-31

**Authors:** In Young Jung, Jung Ju Kim, Se Ju Lee, Jinnam Kim, Hye Seong, Wooyong Jeong, Heun Choi, Su Jin Jeong, Nam Su Ku, Sang Hoon Han, Jun Yong Choi, Young Goo Song, Jung Won Park, June Myung Kim

**Affiliations:** ^1^Department of Internal Medicine, Yonsei University College of Medicine, Seoul, Republic of Korea; ^2^AIDS Research Institute, Yonsei University College of Medicine, Seoul, Republic of Korea; ^3^Institute of Allergy, Yonsei University College of Medicine, Seoul, Republic of Korea

## Abstract

**Background:**

Adverse drug reactions (ADRs) are any unwanted/uncomfortable effects from medication resulting in physical, mental, and functional injuries. Antibiotics account for up to 40.9% of ADRs and are associated with several serious outcomes. However, few reports on ADRs have evaluated only antimicrobial agents. In this study, we investigated antibiotic-related ADRs at a tertiary care hospital in South Korea.

**Methods:**

This is a retrospective cohort study that evaluated ADRs to antibiotics that were reported at a 2400-bed tertiary care hospital in 2015. ADRs reported by physicians, pharmacists, and nurses were reviewed. Clinical information reported ADRs, type of antibiotic, causality assessment, and complications were evaluated.

**Results:**

1,277 (62.8%) patients were considered antibiotic-related ADRs based on the World Health Organization-Uppsala Monitoring Center criteria (certain, 2.2%; probable, 35.7%; and possible, 62.1%). Totally, 44 (3.4%) patients experienced serious ADRs. Penicillin and quinolones were the most common drugs reported to induce ADRs (both 16.0%), followed by third-generation cephalosporins (14.9%). The most frequently experienced side effects were skin manifestations (45.1%) followed by gastrointestinal disorders (32.6%).

**Conclusion:**

Penicillin and quinolones are the most common causative antibiotics for ADRs and skin manifestations were the most frequently experienced symptom.

## 1. Introduction

Adverse drug reactions (ADRs) are any unwanted/uncomfortable effects from medication resulting in physical, mental, and functional injuries [1]. ADRs experienced by hospitalized patients are associated with increased morbidity and mortality, prolonged hospitalization, and increased medical expense [2]. For this reason, several studies have suggested that ADRs are a major public health concern [3].

Disease prevalence, economic status, culture, and ethnicity all contribute to different ADR patterns [4, 5]. The overall incidence of ADRs varies by study but ranges from 0.15% to 30% [1, 6]. In one study conducted at an Indian tertiary care hospital, antibiotics were responsible for 40.9% of ADRs [6]. An Australian tertiary center reported that antibiotics were related to 25% of ADRs [7]. Furthermore, previous studies have shown that 26.88% of ADRs are considered severe and that 99.47% required additional medical intervention [8].

Many observational studies have examined the incidence, pattern, and severity of ADRs, but most of these have been performed in America or Europe; reports on Asian countries are extremely rare [7, 9, 10]. Several South Korean reports have identified antibiotics as a leading cause of ADRs, but most are based on information from primary care center pharmacies, and data on ADRs related to antimicrobial agents reported from tertiary care hospitals are extremely rare.

Although a number of studies on ADRs caused by various drugs have been conducted, none have focused specifically on antibiotics. Therefore, in this study we investigated the frequency of antibiotic-related ADRs experienced at a tertiary health care hospital in South Korea.

## 2. Materials and Methods

### 2.1. Study Design

This was a retrospective cohort study based on reports from Yonsei University College of Medicine Severance Hospital, a tertiary health care hospital in Seoul, South Korea, from January 1 to December 31, 2015. Only antibiotic-related ADRs in hospitalized patients were analyzed. All antibiotics, whether administered concurrently or at a different time point, were evaluated for possibilities of ADRs and included in the analysis. Any cases of ADRs that might have been caused by concurrently administered drugs, other than antibiotics, were excluded from the analysis.

The following data were collected: date of reported ADR, age, gender, clinical manifestation, causal drug and brand name, route of administration, dates of administration and discontinuation, outcome (serious or not serious), recurrence, causality assessment, and dose-relationship. This study was approved by the Institutional Review Board (IRB) of Severance Hospital (IRB #4-2017-0307), and the need for written informed consent from all participants was waived by the approving IRB.

### 2.2. Definitions

Causality was classified into three categories: certain, probable, and possible based on the WHO-Uppsala Monitoring Center criteria [11, 12]. The severity of each ADR was classified as serious or nonserious [12]. Serious ADRs were defined as patients who experienced disability, prolonged hospitalization, life-threatening symptoms, or death [12]. Symptoms were classified according to symptom organ class (SOC) from the Medical Dictionary for Regulatory Activities (MedDRA) [13]. Defined daily dose (DDD) is the average maintenance dose per day for a drug used for its main purpose, as defined by the World Health Organization (WHO) [14]. Antimicrobial use density (AUD) describes the total antimicrobial use in DDD per 1,000 patient days of one drug class, as recommended by the WHO [14]. AUD was calculated as follows.

  AUD = (total antimicrobial use)/(DDD × patient days) × 1,000 [15, 16].

### 2.3. Collected Data and Reporting Sources

Severance Hospital is a 2400-bed tertiary care hospital and is one of the largest health care centers in South Korea. Severance was registered as a Regional Pharmacovigilance Center in 2006 and is using a computer-based pharmacovigilance monitoring system. ADR reporting is voluntary and can be reported by a physician, pharmacist, nurse, or patient who recognizes the ADR event. These voluntary reports are reviewed by the ADR-monitoring team, which includes a physician from the Department of Allergy and Clinical Immunology and a pharmacist. Then the clinical and demographic information of the reported ADR is stored in a pharmacovigilance system database and noted in the patient's electronic medical record (EMR). The computerized system improves medication safety by alerting medical practitioners to drug allergies and any drug-drug interactions the patient experienced.

### 2.4. Data Analysis

Descriptive statistic procedures were performed to analyze the ADR cases. Categorical variables are presented as numbers and percentages. All statistical tests were performed using SPSS 18.0 (Statistical Package for the Social Sciences, Chicago, IL, USA).

## 3. Results

### 3.1. Demographic Data, Severity, and Causality

**Figure 1:**
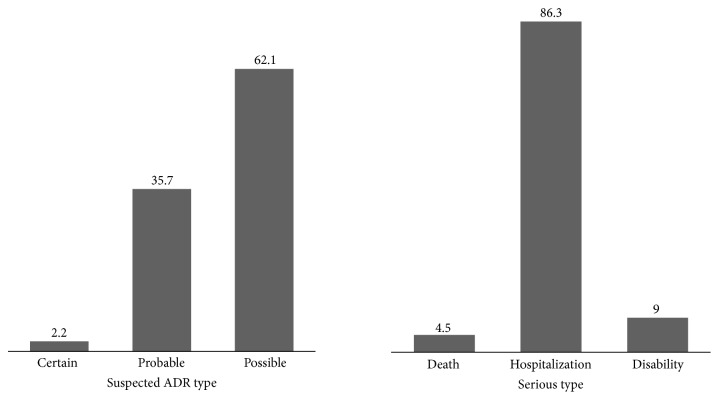
Frequency (%) of adverse drug reaction types and serious adverse drug reactions.

In total, 2,032 cases of antibiotic-related ADRs were reported during the study period. Of these, 1,277 (62.8%) were proven to be antibiotic-related based on the World Health Organization- (WHO-) Uppsala Monitoring Center criteria. The median age was 54 years (range 35–78), and 610 (47.8%) patients were male. Causality assessment based on WHO criteria revealed that 28 (2.2%) cases were certainly caused by antibiotics, 456 (35.7%) were probably caused by them, and 793 (62.1%) were possibly caused by them (Figure 1). A severity assessment confirmed 44 (3.4%) serious ADRs. Death or life-threatening events, hospital admission or prolonged hospital stay, or disability occurred in 2 cases (4.5%), 38 cases (86.3%), and 4 cases (9.0%), respectively.

### 3.2. Frequency of Antibiotic-Related ADRs and Symptoms

Penicillin and quinolones were the most frequent causes of ADRs, and both accounted for 204 cases (16%) (Table 1). Third-generation cephalosporins accounted for 190 cases (14.9%), second-generation cephalosporins accounted for 144 cases (11.3%), and glycopeptides accounted for 134 cases (10.5%).

The most common organ system affected by penicillin was the skin and subcutaneous tissue in 88 cases (43.1%), followed by the gastrointestinal system in 61 cases (29.9%) and immunological system in 22 cases (10.8%). Quinolones also commonly affected the skin and subcutaneous tissue (98 cases, 48%), followed by the gastrointestinal system (66 cases, 32.4%) and the nervous system (16 cases 7.8%). Third-generation cephalosporins resulted in skin and subcutaneous tissue reactions in 86 cases (45.3%), gastrointestinal reactions in 79 cases (41.6), and immunological reactions in 18 cases (9.5%). In particular, immunologic reactions, hypersensitivity (125 cases), anaphylaxis (10 cases), Stevens-Johnson syndrome (2 cases), and angioedema (9 cases) were identified.

### 3.3. Frequency of ADRs by Symptom and the Most Common Causative Antibiotics

**Figure 2:**
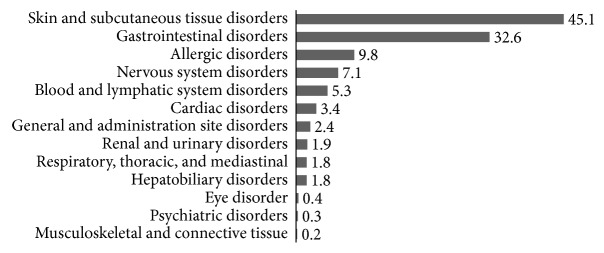
Frequency (%) of adverse drug reaction in symptom organ class.

Skin and subcutaneous tissue disorders were the most common clinical manifestation, occurring in 576 cases (45.1%), followed by gastrointestinal disorders, which occurred in 416 cases (32.6%) (Figure 2).

Quinolones (98 cases, 17%) and penicillin (88 cases, 15.3%) were the most common causative agents for skin and subcutaneous manifestations, followed by third-generation cephalosporins in 86 cases (14.9%) (Table 2). Gastrointestinal disorders were most often caused by third-generation cephalosporins (79 cases, 19.0%), followed by quinolones (66 cases, 15.9%) and penicillin (61 cases, 14.7%).

### 3.4. Antimicrobial Use Density (AUD) to Demonstrate Each Class of Antibiotics Usage

In our study, the antibiotic uses of penicillin were 2,179.2 AUDs, followed by third-generation cephalosporin and quinolone with AUDs of 1,277.8 and 837.9, respectively (Supplementary Table 1).

## 4. Discussion

Several South Korean reports have identified antibiotics as the most common cause of ADRs [17, 18]. However, most of these reports have been based on data from private clinics and pharmacies rather than tertiary care hospitals. Here, we report the antibiotic-related ADRs experienced at a tertiary care hospital.

In this study, 3.4% of patients experienced serious ADRs. One multicenter study conducted in 2009 covering six Regional Pharmacovigilance Centers in South Korea reported that 17.7% of ADRs were serious [18]. A meta-analysis reported that 6.7% of ADRs were serious and that 0.32% of ADRs were fatal [19]. However, it is difficult to compare these results with our study because the previous studies included nonantibiotics such as nonsteroidal anti-inflammatory drugs (NSAIDs) and radiocontrast media.

Antibiotics have been reported to be major causes of ADRs [20]. In a study that only included outpatients, sulfonamides followed by penicillin were reported to be the most common causative antibiotics [20]. Prior reports have shown that quinolones, ciprofloxacin in particular, are another common causative antibiotic [21]. This study shows that penicillin and quinolones were responsible for the majority of ADRs. These results are similar to several other South Korean reports [18, 22].

Geer et al. [6] reported that antituberculosis drugs accounted for 13.15% of all ADRs, and Maciel et al. [23] reported that up to 83.54% of ADRs were caused by antituberculosis drugs. In a study in Iran, gastrointestinal symptoms (22%) and hepatotoxicity (35.7%) were frequently experienced ADRs caused by antituberculosis drugs [24]. In this study, antituberculosis medications made up a smaller proportion (61 cases 4.8%) of ADRs; however, gastrointestinal reactions (11.5%) and hepatotoxicity (9.8%) were both common symptoms experienced in our study, which is similar to the results of previous studies. Isoniazid was accountable for nausea/vomiting in 2 cases, hepatobiliary disorders in 4 cases, and skin and subcutaneous tissue disorders in 8 cases, and 1 case was associated with anaphylaxis. Rifampin was accountable for nausea/vomiting, skin and subcutaneous tissue disorders, and allergic disorders in 3, 9, and 4 cases, respectively. 1 case was associated with rifampin induced Stevens-Johnson syndrome. Ethambutol ADRs were mostly associated with skin and subcutaneous tissue disorders (13 cases), and ethambutol induced optic neuritis was confirmed in 4 cases. The majority of pyrazinamide ADRs were also skin and subcutaneous tissue disorders, 5 cases.

Of all cutaneous ADRs considered in a previous study, antibiotics were the main cause (46.55%) [25]; in another study, antibiotics accounted for 48% of delayed cutaneous ADRs, 20% of which were purportedly due to glycopeptides and sulfonamides [26]. In particular, glycopeptides and sulfonamides were implicated in 20% of these ADRs [26]. In our study, 45.1% of skin and soft tissue ADRs were due to antimicrobial agents. Quinolones, penicillin, third-generation cephalosporins, and glycopeptides were the most common causative antibiotics for skin and subcutaneous-related ADRs. The difference in causative antibiotics may be explained by the ethnicities included in each study [5]. Further studies on the mechanisms behind causative antibiotics and reactions are needed.

Penicillin allergies are more common in females [27], as is the frequency of ADRs [28]. We also found a slight female predominance in our study (47.8% of patients who experienced ADRs were male).

There were several limitations to our study. First, it was a single-center study and lacked reports from private clinics and other Asian countries. Further studies regarding antibiotics and ADRs are necessary to validate our results and provide more generalizable data covering all Asian countries. Second, reports of ADRs are voluntary at our hospital, so many cases could have gone unreported. Third, only data on hospitalized patients were collected; ADRs of outpatients were not included in the study. Finally, DDD is a unit of measurement and does not necessarily reflect the recommended dose or prescribed daily dose (PDD). The PDD for each class of antibiotic was not reported by the pharmacovigilance monitoring system used in this study. As there is a known discrepancy between the PDD and the daily DDD, further validation by PDDs would be necessary for accurate comparisons between antibiotics.

## 5. Conclusions

In conclusion, penicillin and quinolones were the most common antibiotic causes of ADRs. The most frequently experienced clinical feature was skin manifestations. These findings may help identify patterns and causative antibiotics of ADRs in Asian countries.

## Figures and Tables

**Table 1:** Antibiotics related ADR frequency and most common symptoms according to organ class (according to the preferred terms of MedDRA coding system).

Antibiotic	Patients, *n* (%)	Symptom organ class	Frequency of ADRs, *n* (%)
Penicillin	204 (16)	Skin and subcutaneous tissue Gastrointestinal Allergic	88 (43.1) 61 (29.9) 22 (10.8)

Quinolone	204 (16)	Skin and subcutaneous tissue Gastrointestinal Nervous system	98 (48.0) 66 (32.4) 16 (7.8)

3rd cephalosporin	190 (14.9)	Skin and subcutaneous tissue Gastrointestinal Allergic	86 (45.3) 79 (41.6) 18 (9.5)

2nd cephalosporin	144 (11.3)	Skin and subcutaneous tissue Gastrointestinal Nervous system	68 (47.2) 53 (36.8) 12 (8.3)

Glycopeptide	134 (10.5)	Skin and subcutaneous tissue Allergic Blood and lymphatic system	83 (61.9) 24 (17.9) 14 (10.4)

Metronidazole	61 (4.8)	Gastrointestinal Skin and subcutaneous tissue Nervous system	46 (75.4) 12 (19.7) 5 (8.2)

Antituberculosis medication	61 (4.8)	Skin and subcutaneous tissue Gastrointestinal Hepatobiliary Allergic	33 (54.1) 7 (11.5) 6 (9.8) 6 (9.8)

1st cephalosporin	53 (4.2)	Skin and subcutaneous tissue Nervous system	32 (60.4) 18 (34.0) 6 (11.3)

Carbapenem	43 (3.4)	Skin and subcutaneous tissue Allergic Gastrointestinal	19 (44.2) 10 (23.3) 8 (18.6)

Antifungal	33 (2.6)	Allergic Skin and subcutaneous tissue Cardiac	12 (36.4) 9 (27.3) 6 (18.2)

Antiviral	21 (1.6)	Skin and subcutaneous tissue Gastrointestinal Blood and lymphatic system	8 (38.1) 5 (23.8) 3 (14.3)

Aminoglycoside	20 (1.6)	Skin and subcutaneous tissue Gastrointestinal Renal and urinary	12 (60.0) 4 (20.0) 2 (10.0)

Macrolide	17 (1.3)	Gastrointestinal Skin and subcutaneous tissue Nervous system	7 (41.2) 5 (29.4) 3 (17.6)

Sulfonamide	16 (1.3)	Gastrointestinal Skin and subcutaneous tissue Renal and urinary	9 (56.2) 5 (31.2) 2 (12.5)

4th cephalosporin	16 (1.3)	Skin and subcutaneous tissue Gastrointestinal Nervous system	9 (56.2) 5 (31.2) 3 (18.8)

Tetracycline	13 (1)	Gastrointestinal Skin and subcutaneous tissue	8 (61.5) 2 (15.4)

Antimalarial	12 (0.9)	Skin and subcutaneous tissue Gastrointestinal Nervous system	5 (41.7) 4 (33.3) 4 (33.3)

Lincosamide	9 (0.7)	Skin and subcutaneous tissue Gastrointestinal	7 (77.8) 2 (22.2)

Polymyxin	3 (0.2)	Renal and urinary Skin and subcutaneous tissue	2 (66.7) 1 (33.3)

Monobactam	1 (0.1)	Allergic Skin and subcutaneous tissue	1 (100) 1 (100)

Linezolid	1 (0.1)	Blood and lymphatic system	1 (100)

ADRs: adverse drug reactions.

**Table 2:** Frequency of ADRs in symptom organ class and most common causative antibiotics (according to the preferred terms of MedDRA coding system).

Symptom organ class	Patients, *n* (%)	Antibiotics	Frequency of ADRs, *n* (%)
Skin and subcutaneous tissue disorders	576 (45.1)	Quinolone Penicillin 3rd cephalosporin	98 (17.0) 88 (15.3) 86 (14.9)

Gastrointestinal disorders	416 (32.6)	3rd cephalosporin Quinolone Penicillin	79 (19.0) 66 (15.9) 61 (14.7)

Allergic disorders	125 (9.8)	Glycopeptide Penicillin 3rd cephalosporin	24 (19.2) 22 (17.6) 18 (14.4)

Nervous system disorders	91 (7.1)	Quinolone 3rd cephalosporin 2nd cephalosporin	16 (17.6) 14 (15.4) 12 (13.2)

Blood and lymphatic system disorders	68 (5.3)	Penicillin Glycopeptide 3rd cephalosporin	20 (29.4) 14 (20.6) 8 (11.8)

Cardiac disorders	43 (3.4)	Quinolone 3rd cephalosporin 2nd cephalosporin	8 (18.6) 7 (16.3) 7 (16.3)

General disorders and administration site conditions	31 (2.4)	Quinolone Antiviral agent	11 (61.1) 2 (11.1)

Renal and urinary disorders	24 (1.9)	Glycopeptide Penicillin Antifungal agent	5 (20.8) 5 (20.8) 3 (12.5)

Respiratory, thoracic, and mediastinal disorders	23 (1.8)	3rd cephalosporin Antifungal agent Penicillin	6 (26.1) 5 (21.7) 4 (17.4)

Hepatobiliary disorders	23 (1.8)	Anti-TB medication Penicillin Carbapenem	6 (26.1) 3 (13.0) 3 (13.0)

Eye disorder	5 (0.4)	Anti-TB medication Penicillin	4 (80.0) 1 (20.0)

Psychiatric disorders	4 (0.3)	2nd cephalosporin Carbapenem Quinolone	2 (50.0) 1 (25.0) 1 (25.0)

Musculoskeletal and connective tissue disorders	2 (0.2)	Penicillin Antifungal agent	1 (50.0) 1 (50.0)

ADRs: adverse drug reactions; TB: tuberculosis.

## Supplementary Materials

Supplementary Materials Supplementary Table 1. Uses of antibiotics during study period, describing the antimicrobial use density (AUD) for each class of antibiotics to demonstrate antibiotics usage during the study period. AUD was a defined daily dose (DDD) per 1,000 patient days. DDD is the average maintenance dose per day for a drug used for its main purpose, as defined by the World Health Organization (WHO). AUD was calculated as (total antimicrobial use)/(DDD × patient days) × 1,000 with reference to prior studies.
