# Supplementary material for: Antibiotic-Related Adverse Drug Reactions at a Tertiary Care Hospital in South Korea
Source: Biomed Res Int. 2017 Dec 31;2017:4304973. doi: 10.1155/2017/4304973 (PMC5804292; doi:10.1155/2017/4304973)
Supplement: Supplementary Materials — Supplementary Table 1. Uses of antibiotics during study period, describing the antimicrobial use density (AUD) for each class of antibiotics to demonstrate antibiotics usage during the study period. AUD was a defined daily dose (DDD) per 1,000 patient days. DDD is the average maintenance dose per day for a drug used for its main purpose, as defined by the World Health Organization (WHO). AUD was calculated as (total antimicrobial use)/(DDD × patient days) × 1,000 with reference to prior studies. [file 4304973.f1.docx]

Supplementary table 1. Antibiotic uses of antibiotics during study period

| Antibiotic | Antibiotic uses (AUD) |
| --- | --- |
| Penicillin | 2179.2 |
| 3^rd^ cephalosporin | 1277.8 |
| Quinolone | 837.9 |
| Carbapenem | 817.4 |
| Glycopeptide | 787.6 |
| 2^nd^ cephalosporin | 722.8 |
| Sulfonamide | 367.9 |
| Aminoglycoside | 347.2 |
| 4^th^ cephalosporin | 341.7 |
| Antifungal | 300.9 |
| 1^st^ cephalosporin | 276.1 |
| Metronidazole | 246.8 |
| Polymyxin | 79.0 |
| Lincosamide | 47.8 |
| Tetracycline | 43.8 |
| Macrolide | 34.6 |
| Linezolid | 30.0 |
| Monobactam | 18.7 |

AUD; antimicrobial use density

AUD was a defined daily dose (DDD) per 1,000 patient days
